# Perspectives on the expansion of human precision oncology and genomic approaches to sea turtle fibropapillomatosis

**DOI:** 10.1038/s42003-019-0301-1

**Published:** 2019-02-07

**Authors:** David J. Duffy, Mark Q. Martindale

**Affiliations:** 1 Department of Biological Sciences, School of Natural Sciences, Faculty of Science and Engineering, University of Limerick, Limerick, V94 T9PX Ireland; 2 The Whitney Laboratory for Marine Bioscience and Sea Turtle Hospital, University of Florida, St. Augustine, FL 32080 USA

## Abstract

Our recent Communications Biology research article revealed the genomic drivers and therapeutic vulnerabilities of sea turtle fibropapillomatosis tumors. Fibropapillomatosis is a debilitating tumorous disease afflicting populations of green sea turtles globally. While a virus is involved in the development of this disease, it is increasingly understood that the key trigger is linked to anthropogenic disturbances of the environment. The specific environmental co-trigger(s) has yet to be functionally confirmed. Here we outline the next steps required to advance our understanding of this enigmatic disease, to enable us to more effectively clinically combat it and to ultimately tackle its environmental co-trigger to halt and hopefully reverse the spread of fibropapillomatosis.

## Introduction

**Fig. 1:**
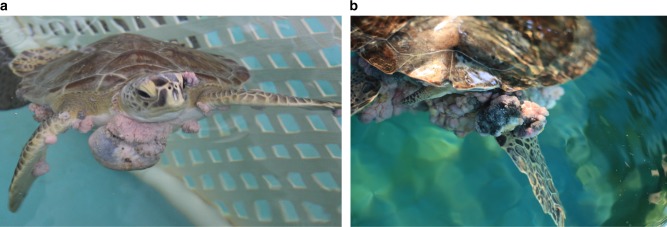
Fibropapillomatosis-afflicted green sea turtles (*C. mydas*). Anterior (**a**) and posterior (**b**) view of fibropapillomatosis tumors on juvenile green sea turtles prior to tumor removal surgery at the University of Florida’s Whitney Laboratory Sea Turtle Hospital. Right image used under creative commons license, CC BY-ND (https://theconversation.com/could-human-cancer-treatments-be-the-key-to-saving-sea-turtles-from-a-disfiguring-tumor-disease-98140)

As the risk of extinction of a species increases, so, too, does the effect of diseases on the population of that species^1,2^. This can create a snowball effect whereby emerging pathogens potentially accelerate the extinction of the species. Given the dire rate of habitat loss, biodiversity decline and unparalleled human-induced species loss (on a par with previous mass extinction events)^3–7^, it is imperative that we are increasingly vigilant in relation to endangered wildlife disease outbreaks. Indeed, human activities might be contributing to oncogenic processes in wild animal populations^8,9^. A host of tumorous diseases affecting wildlife populations are pathogen-induced^10,11^, as are at least 15% of human cancers^12,13^. One such pathogen-induced tumorous disease is sea turtle fibropapillomatosis, a virulent global epizootic (animal epidemic) leading to tumors on turtles’ soft tissues and internal organs (Fig. 1). Individual turtles are often severely afflicted by tens to hundreds of tumors which can lead to fatalities. The myriad other anthropogenic threats that endangered sea turtle species already face (IUCN Red List, https://www.iucnredlist.org/) means they can ill afford to also combat this disease. Our ability to manage, prevent and treat fibropapillomatosis is severely hampered by a lack of knowledge of the disease’s triggers and the genomic events driving tumor formation and growth.

Fibropapillomatosis afflicts all seven sea turtle species, of which green sea turtles (*Chelonia mydas*) is the most severely afflicted^14–16^. The number of stranded *C. mydas* exhibiting fibropapillomatosis has dramatically increased in recent years, within Florida and globally^14,15,17^. Fibropapillomatosis is ideally suited to in-depth molecular and clinical study given the large numbers of patients treated in rehabilitation facilities annually^1,14,16,18^.

Our application of cutting-edge genomic and precision medicine approaches to fibropapillomatosis research has already advanced sea turtle conservation medicine and provided novel insights into viral-environmental oncogenic interactions applicable to human medicine^1,14,18^. Deep-sequencing-based approaches are enabling dramatic advances^1,19,20^ beyond earlier fibropapillomatosis gene expression research^21^. We revealed the genomic drivers and therapeutic vulnerabilities of sea turtle fibropapillomatosis in our recent Communications Biology article^14^. The global importance of this disease^16^, combined with the urgent need to modernize conservation medicine^1^, and the potential insights this sea turtle tumor epidemic holds for human environmental and pathogen-induced tumors^1,14^ has prompted us to greatly expand the scope of our fibropapillomatosis research. Here we discuss the advances required to better understand and tackle this enigmatic conservation-relevant disease. Our ongoing research endeavors have the combined goals of seeking to improve sea turtle conservation medicine and survival, enhancing our fundamental understanding of this tumorous disease, and advancing our comprehension of how detrimental environmental-pathogen-host interactions can drive wildlife and human tumorigenesis.

### Tumor genomic & transcriptional profiling (host & virus) and enhanced therapeutic target identification

Highlighting the power of modern genomic and precision medicine techniques, the transcriptomic profiling that formed the core of our recent Communications Biology paper^14^ was performed using only seven fibropapillomatosis tumor samples and three non-tumored skin control samples (patient-matched). Despite this low sample size we were able to gain important insight into the molecular mechanisms driving fibropapillomatosis tumors, identify the transcriptionally latent state of the fibropapilloma-associated pathogen, Chelonid herpesvirus 5 (ChHV5) within established tumors, and determine promising anti-fibropapillomatosis drug treatments. However, many mysteries still remain in relation to this disease. Therefore, we have greatly increased the number of tumors (and control samples) transcriptomically profiled in order to refine our drug target selection and answer a range of questions in relation to the environmental, host and viral drivers of this disease. We are particularly interested in improving our genomic understanding of early-stage tumors, post-surgical regrowth tumors and internal tumors.

Understanding the genetic relationship between the numerous tumors occurring on individual turtles will have implications for the clinical treatment of the disease, as well as providing insight into the initiating mechanisms of the tumors. We are actively investigating whether these tumors are clonal (i.e. metastatically spread) or if each tumor arises de novo (primary tumors) and has unique mutational profile and impact on distinct tissue types. We are also implementing detailed tumor (re)growth profiling to more robustly assess growth dynamics and the effect of therapeutic treatments^18^.

In terms of novel therapeutic approaches, because the vast majority of patients are simultaneously afflicted by tens or more tumors, we are prioritizing the identification of orally deliverable compounds to achieve systemic responses. We are also specifically seeking to identify therapeutic compounds for the treatment of internal tumors, which are currently inoperable. Internal tumors most commonly afflict the lungs, kidneys, heart, gastrointestinal tract, and liver^22^. Currently no treatment exists for turtles with visceral organ tumors, as the hard external shell (plastron and carapace) hampers surgical access to fibropapillomatosis-afflicted organs. Patients diagnosed with internal tumors are instead humanely euthanized, often after months of rehabilitation effort has already been expended.

In addition to determining host gene expression essential for the maintenance and growth of fibropapillomatosis tumors it will be crucial to further characterize the expression of ChHV5. We are currently determining the abundance and differential expression of ChHV5 viral transcripts across established tumors, early-stage tumors, post-surgical regrowth tumors, and internal tumors. Furthermore, we are employing unbiased deep-sequencing approaches to compare viral transcript expression levels with the overall level of ChHV5 DNA present within each tumor. Previous approaches to investigate ChHV5 load have primarily relied upon DNA-based PCR or qPCR of a small number of ChHV5 assays or targeted ChHV5 genome sequencing (e.g. enrichment of ChHV5 DNA by long-range PCR amplification)^23–25^.

Together this research promises to shed further light on the viral and host molecular mechanisms responsible for the initiation, maintenance and regrowth of fibropapillomatosis tumors, enabling the design of rational treatment strategies.

### Prognostic biomarkers and viral shedding

We are exploring the potential of prognostic and diagnostic biomarkers to improve the clinical care of fibropapillomatosis-afflicted sea turtles. Currently, x-ray, endoscopy, magnetic resonance imaging (MRI), or computed tomography (CAT) scans are required to diagnose internal tumors. To overcome their limitations in hard-shelled patients, including difficulty in detecting early-stage tumors, we are researching the diagnostic and prognostic value of qPCR-based blood-plasma biomarkers. We are investigating putative biomarkers for both the detection of internal tumors, and biomarkers capable of predicting ultimate rehabilitation outcome of individual turtles. Circulating tumor DNA in blood is a well-established source material for blood-based biomarkers for cancer detection, and is increasingly utilized in human oncology^26–28^.

We are also utilizing pioneering environmental DNA (eDNA) screening techniques to quantify the level of ChHV5 viral shedding in fibropapillomatosis-afflicted sea turtles. eDNA is an ultra-modern approach, whereby free DNA is isolated directly from an environmental sample (e.g. cellular material from water) and subsequently detected by molecular approaches^29–31^ (e.g. deep sequencing or qPCR). By tracking the level of virus shed into the environment by each patient, we will better understand disease transmission dynamics. Furthermore, by correlating viral shedding to individuals throughout the various stages of the rehabilitation process, and individuals with varying tumor burdens and tumor aggressiveness^18^ we can begin to quantify whether there are certain times/states which promote enhanced viral shedding. Such profiling can also help inform clinical management (e.g. isolation of high rate viral shedders), and potentially improve our understanding of how this disease spreads throughout the marine environment.

### Dynamics of environmental fibropapillomatosis disease origins

While ChHV5 is likely a key driver of fibropapillomatosis, additional environmental co-triggers are required to drive fibropapillomatosis tumor formation in a laboratory setting^32^ and in wild populations. A number of potential environmental co-triggers have been investigated, some of which correlate strongly with the disease incidence. However, no factor has yet been causatively proven to drive fibropapillomatosis tumor development^14,16^.

While there are a number of putative environmental triggers that warrant further investigation, the correlation between elevated ultraviolet (UV) radiation levels and disease incidence, as well as the shared molecular drivers of fibropapillomatosis and human basal cell carcinoma^14^ suggest a possible role of UV radiation as an environmental co-trigger of fibropapillomatosis. This includes the differential regulation of Wnt and Metalloprotease signaling^14^ in fibropapillomatosis. These pathways are known to be regulated by UV exposure in human skin cells and canine cornea^33,34^. UV exposure has been linked to systemic immunosuppression^35,36^, which also occurs in turtles with fibropapillomatosis, although it is not yet determined whether this is a cause or a consequence of the disease^16^. Elevated UV exposure therefore holds the potential to induce immunosuppression in turtle skin, possibly enabling the rampant proliferation of ChHV5 and the crossing of an oncogenic threshold.

**Fig. 2:**
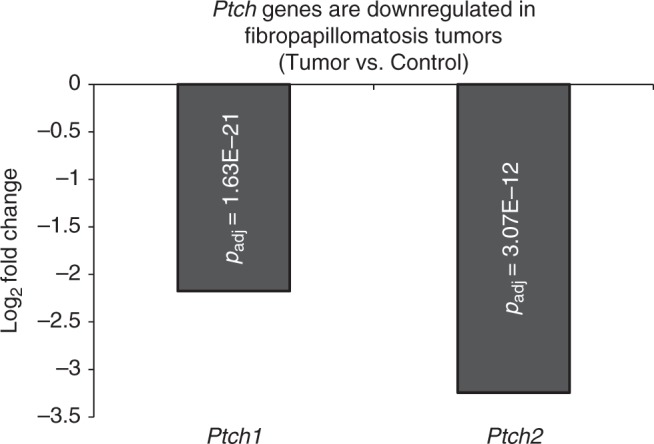
Downregulation of *Patched* (*Ptch*) tumor suppressor genes in fibropapillomatosis tumors, as revealed by RNA-seq, adapted from Duffy et al.^14^

UV exposure is also a known tumorigenic mutagen^37^. Fibropapillomatosis tumors primarily occur on external regions, suggesting UV light may act as a direct mutagen contributing to oncogenesis, like with other human and wildlife skin cancers. UV exposure induces signature mutations in genes such as *p53* (*Tp53*) and *Patched* (*Ptch*), two tumor suppressor genes responsible for human non-melanoma skin cancer^37^. Sunlight also acts as a tumor promoter by favoring clonal expansion of such mutated cells^37^. Expression of both *Ptch* genes are highly suppressed in fibropapillomatosis tumors^14^ (Fig. 2). Mutational analysis of tumor genomes is now required to confirm whether this is due to UV-induced mutations. We have begun to investigate the presence of mutations within fibropapillomatosis tumors, focusing primarily on UV-induced signatures. This approach^38–40^ will also identify any other mutation-inducing exogenous exposures responsible for driving fibropapillomatosis tumorigenesis.

Juvenile *C. mydas*, the life-stage afflicted by fibropapillomatosis, reside in shallower UV-exposed tropical and sub-tropical inshore waters, while the predominantly tumor free post-hatchlings, sub-adults, and adults dwell in the open water^16^. A role of UV in the disease’s etiology would help to explain the unusual dynamics of its geographic spread. Fibropapillomatosis took over 80 years to spread from the Florida Keys (first recorded site of FP incidence globally^41,42^) to northern Florida, a distance of ~450 miles, despite neighboring juvenile green turtle populations running the length of the entire seaboard. Conversely, in a shorter time period the disease spread to distant *C. mydas* populations circum-globally, throughout equatorial and sub-equatorial zones^15^. UV levels reaching the Earth’s surface have increased in recent times both locally in Florida^14^ and globally^43^, potentially driving the spread of the disease away from the equator and increasing incidence in some longer-affected areas.

UV might also indirectly contribute to tumourigenesis by converting inshore chemicals into more potentent oncogenic forms. UV light is known to degrade organic compounds in natural waters (photolysis). Complex organic (often poly-unsaturated/aromatic) molecules can be broken down into smaller, more reactive compounds that can then react with each other forming different, potentially harmful compounds^44,45^. While this process is globally ubiquitous, matching fibropapillomatosis incidence, it is most prominent in tropical and subtropical latitudes where sunlight is most intense and in areas where organic compound concentrations are highest (nearshore and estuarine waters). Therefore, this offers another potential mechanism through which UV could be indirectly contributing to fibropapillomatosis initiation.

Whether fibropapillomatosis tumourigenesis is triggered by UV directly (induction of genomic mutations) or indirectly (UV-induced immunosuppression or UV-induced conversion of inshore chemicals), with the increases in UV exposure occurring globally it is likely that mechanistic findings will be highly relevant not only for sea turtle fibropapillomatosis but also other human and wildlife oncogenic diseases.

### Human and wildlife precision medicine: mutually beneficial crosspollination

Fibropapillomatosis provides an ideal opportunity to study pathogen and environmentally co-induced tumors. This will improve our ability to combat not only this sea turtle disease epizootic but also the ever-increasing number of wildlife and human cancers discovered to be pathogen-induced (over 15% of human cancers^13^) or exacerbated by pathogens^46,47^. The key role of environmental co-triggers further complicates our understanding of pathogen-induced cancers. In humans, studying the initiating events that can go on to produce later cancers is difficult. Once assumed to be an issue for vulnerable cohorts (e.g. Kaposi sarcoma-associated herpesvirus drives tumor formation in immunocompromised patients)^12^, it is now understood that temporally distant viral events can sow the seeds of later cancers (e.g. human papilloma virus [HPV] infection can ultimately result in cervical and throat cancers^48^). HPV is also clinically associated with human non-melanoma skin cancer, including basal cell carcinoma^48–50^ which is the cancer type that transcriptomically most closely resembles chelonid fibropapillomatosis^14^. Furthermore, UV radiation and HPV infection are linked to human non-melanoma skin cancer, with UV-induced immunosuppression being a likely driving mechanism^35,51^. We urgently need tractable natural models of virally induced tumors to fully understand the role of pathogens in driving tumor formation and to determine environmental co-initiation mechanisms^52^. Sea turtles have a similar life-span to humans and have normally robust anti-cancer defenses, providing a more faithful comparative model of humans than common short-lived cancer models, such as rodents and zebrafish^1^. Fibropapillomatosis offers an intriguing new glimpse into a potentially devastating disease mechanism, revealing that an environmental co-factor is potent enough to enable a normally benign virus to cause disease of epidemic (epizootic) proportions in otherwise healthy juvenile populations. Intensive study of chelonid fibropapillomatosis will enable us to directly address the crucial question of how an environmental trigger is capable of conferring such potent oncogenicity on a clinically benign virus, an issue that potentially holds immense importance for our understanding of human and wildlife pathogen-induced tumors.

### Future outlook

Interesting work is emerging regarding green turtle cell culture and the enigmatic ChHV5 virus^23,53,54^. Importantly, the breakthrough enabling the long sought-after ability to culture the virus in the lab means that Koch’s postulates (four criteria required to identify the causative agent of an infectious disease) can hopefully be fully assessed in the near future^53^. If Koch’s postulates are fulfilled, ChHV5 can finally be comprehensively claimed as the primary pathogenic agent driving tumor formation, albeit possibly still requiring environmental co-trigger(s) to unleash sufficient viral loads. Alternatively, if not fulfilled this will demonstrate that ChHV5 inoculation/exposure alone truly is not sufficient to drive this disease and that another trigger(s)/co-trigger(s) is essential to initiate fibropapillomatosis tumor growth. While it is possible that an alternative pathogen might be identified, the correlation of ChHV5 viral load to tumors and the lack of other pathogens in significant abundance within the tumors^16^ makes this less likely. The lack of a specific tumor associated virulent ChHV5 strain and the presence of ChHV5 in tumor-free populations^16^ demonstrate that the limiting factor in developing fibropapillomatosis is not just exposure to ChHV5 but also exposure to the elusive inshore environmental co-trigger(s).

Advances in cell biology, environmental oncogenic trigger detection and assessment, and genomic medicine mean that now more than ever we are within sight of revealing the longstanding mysteries of the fibropapillomatosis disease epizootic. For our part, we will continue to pioneer advanced genomic and precision wildlife medicine approaches aiming to better understand, treat and ultimately prevent the burden of fibropapillomatosis on sea turtle populations.
